# Plant defense under Arctic light conditions: Can plants withstand invading pests?

**DOI:** 10.3389/fpls.2022.1051107

**Published:** 2022-11-24

**Authors:** Axel Mithöfer, Michael Riemann, Corine A. Faehn, Anna Mrazova, Laura Jaakola

**Affiliations:** ^1^ Research Group Plant Defense Physiology, Max Planck Institute for Chemical Ecology, Jena, Germany; ^2^ Botanical Institute, Karlsruhe Institute of Technology, Karlsruhe, Germany; ^3^ Department of Arctic and Marine Biology, The Arctic University of Norway, Tromsø, Norway; ^4^ Institute of Entomology, Biology Centre of Czech Academy of Science, Ceske Budejovice, Czechia; ^5^ Faculty of Science, University of South Bohemia, Ceske Budejovice, Czechia; ^6^ NIBIO, Norwegian Institute of Bioeconomy Research, Ås, Norway

**Keywords:** climate change, pest distribution, plant defense, jasmonate signaling, light regime

## Abstract

Global warming is predicted to change the growth conditions for plants and crops in regions at high latitudes (>60° N), including the Arctic. This will be accompanied by alterations in the composition of natural plant and pest communities, as herbivorous arthropods will invade these regions as well. Interactions between previously non-overlapping species may occur and cause new challenges to herbivore attack. However, plants growing at high latitudes experience less herbivory compared to plants grown at lower latitudes. We hypothesize that this finding is due to a gradient of constitutive chemical defense towards the Northern regions. We further hypothesize that higher level of defensive compounds is mediated by higher level of the defense-related phytohormone jasmonate. Because its biosynthesis is light dependent, Arctic summer day light conditions can promote jasmonate accumulation and, hence, downstream physiological responses. A pilot study with bilberry (*Vaccinium myrtillus*) plants grown under different light regimes supports the hypothesis.

## Introduction

Steadily rising worldwide greenhouse gases emissions have caused an increase of the global temperature of >1 °C within the last 50 years (IPCC, 2021). The predicted global warming is likely to lead to extreme weather events such as tropical storms, extreme precipitations, flooding events, rise of sea level, extreme heatwaves interspersed with drought periods, and many more with huge negative impacts on human societies, the environment and, in particular, on agriculture (see: Cushman et al., 2022, and reviews therein). For example, for temperate zones, an increase of crop loss to herbivorous insects by 10% – 25% per degree Celsius is predicted (Deutsch et al., 2018). As another consequence of the ongoing climate change, temperature in high-latitude regions is rising twice as fast as elsewhere (IPCC, 2019). Due to such warming, Northern climates are becoming increasingly susceptible to expanded insect pest invasion and distributions causing disease emergence and transmission (Bebber et al., 2013; Chaloner et al., 2021). This means climate change may provide the environmental conditions required for the emergence of existing pathogens or the introduction of new pathogens as major threats (Delgado-Baquerizo et al., 2020). Although still speculative, projections anticipate a general correlation of increasing latitudinal distribution with mean global temperature, due to the availability of host plants or through direct climate change effects on the pests (Bebber et al., 2013; Bebber, 2015). Higher night temperatures and milder winters may enable increased winter survival rates of pathogens, insect vectors, and insect herbivores (Harvell et al., 2002). This may also increase the possibility of pest outbreaks. In any case, it will result in reduced plant growth and losses in crop yields.

This scenario will also severely affect Subarctic and Arctic (>60° N) ecosystems, for example by changing the distribution of species and their interactions (Wookey et al., 2009), as well as agriculture. Due to climatic changes, some regions of the Arctic are being used for agricultural purposes since the 1990s to produce potatoes, berries, and herbs among other crops (Leskien, 2020; Mølmann et al., 2021). This holds true in particular for Norway with relative higher temperatures in the Arctic (>66° N) region, due to the Gulf stream, facilitating the growth of various crops even on Svalbard (Spitsbergen, between 74° and 81° N) (Leskien, 2020). However, for the Northern hemisphere, important groups of microbial pathogens (Bacteria, Fungi, Oomycetes), and pests (insects: Coleoptera, Diptera, Hemiptera, Isoptera, Lepidoptera; mites: Acari) showed an increase in Northbound movements since 1960 with an average speed of 2.7 km year^-1^ (Bebber et al., 2013). Thus, it can be speculated that with increasing temperatures and thawing permafrost soil, pathogens and pests will meet naturally occurring plants and crops in Arctic climates in the future. Certainly, not all organisms adapt to climate change at the same speed (Buras and Menzel, 2019; Vindstad et al., 2022). For example, increasing temperature shifts lepidopteran larvae populations towards the Northern regions faster than plant populations move (e.g., Ayres and Scriber, 1994; Hellmann et al., 2008; Batalden et al., 2014), and locally well adapted herbivores are often poorly adapted elsewhere (Maron et al., 2019) but for sure adapt faster than locally adapted host plants. However, many organisms have demonstrated phenotypic plasticity to these challenges through the range, phenological and behavioral shifts, although genetic adaptations can take several generations (Agrawal, 2001). Thus, very likely widespread damage to vegetation will negatively affect plant growth and crop yield and reduce the few positive effects of warming in Northern latitudes.

Nevertheless, there are some reports showing that climate change may strengthen plants. A study with microbial bio-protectants showed that the presence of a plant growth-promoting bacterium (*Acidovorax radices* N35) in the soil increased crop (barley, *Hordeum vulgare*) growth and simultaneously reduced insect pests (aphids, *Sitobion avenae*) under environmental condition simulating climate change-related elevated ozone (O_3_) levels (Zytynska et al., 2020). This demonstrates the potential of local species interactions in mitigating climate change impacts on plants. The authors speculate that under the selected conditions the bacteria induced systemic plant defense by altering plant hormone signaling (Zytynska et al., 2020). A recent study of the invasive plant *Ambrosia artemisiifolia* under the experimental combination of both warming and biocontrol by an herbivorous beetle (*Ophraella communa*), showed that at ambient temperatures-increased resistance was costly, whereas warming promoted better defended plants (Sun et al., 2022). This indicates that on the one hand, invasive plants may be more difficult to control under climate warming due to increased resistance to herbivory; on the other hand, warming may support the natural defensive ability of many other plants.

**Figure 1:**
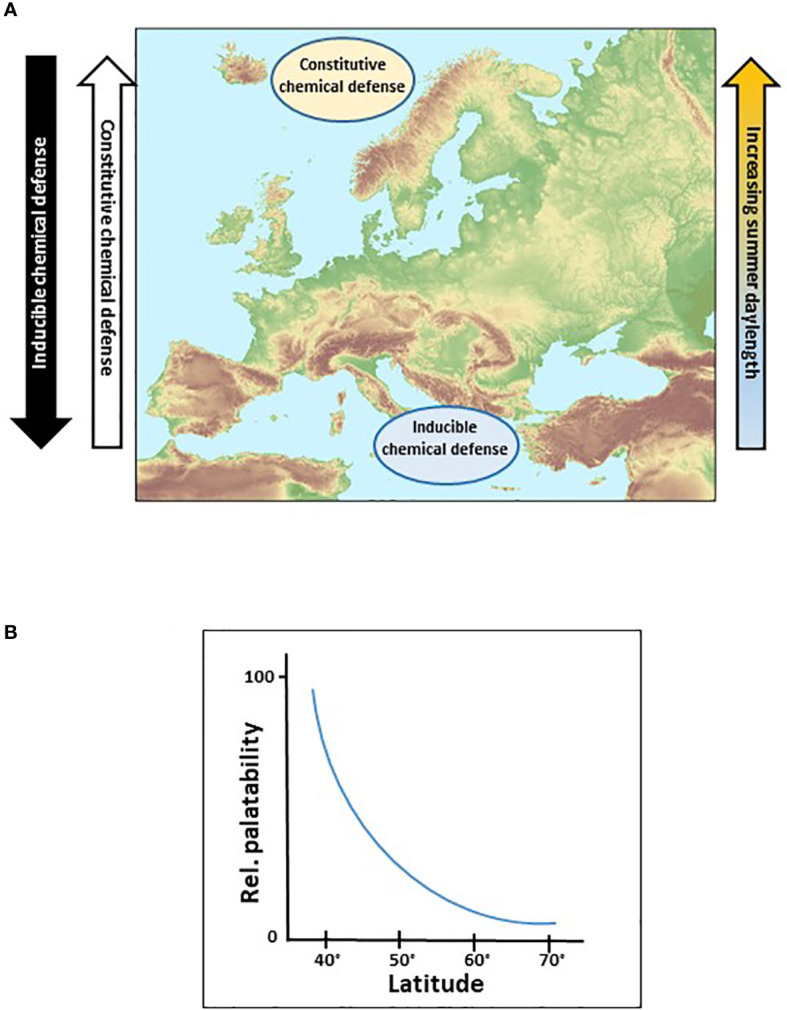
Hypothetic distribution of plant chemical defenses along latitudinal gradients. **(A)** Variability of chemical defenses along latitudinal and their corresponding daylength gradients. **(B)** Proposed decrease of palatability for herbivorous insects feeding on leaves with increasing latitude. © Map of Europe in **(A)** European Environment Agency (EEA).

Plant protection against pathogens and herbivores may be either constitutively present (e.g., morphological features, colour, secondary metabolites) or induced upon stress (e.g., semiochemicals, herbivore-induced plant volatiles, trichomes) (Mithöfer and Boland, 2012). While synthesis of constitutive defensive traits is costly, inducible defenses, in contrast, are triggered only on demand and are therefore relatively less expensive. It is well accepted that growth and defense strategies of plants are evolutionarily intertwined. Investment in defense is assumed to compete with growth for resources (Woods et al., 2012); however, plant growth rate has been predicted to evolve in concert with the resource availability and abiotic factors, which is thought to set the template for defensive adaptations (Coley et al., 1985). Because the replacement costs for tissues is higher for slower-growing plants, they are thought to invest in higher levels of defense compared with faster-growing plants (Woods et al., 2012). Latitude may well be a factor influencing the trade-off between plant defense mechanisms in opposite directions: inducible plant defense correlates negatively and constitutive defense correlates positively with increasing latitudes (**Figure 1A**). In general, herbivore pressure is higher in the South but the nature of the selective pressure is intrinsically different between higher and lower latitudes and an increase in herbivore density with decreasing latitude does not necessarily create a corresponding damage gradient (Salazar and Marquis, 2012). The trade-off between inducible and constitutive defense could occur along a gradient, with strong expressions in the South and the far North (**Figure 1A**). This hypothesis is supported by the fact that plant biomass in the North is less regenerative (De Frenne et al., 2012), grows slowly (Urban et al., 1993), and herbivory is reduced (Rasmann et al., 2014) suggesting a high level of defense compounds. This implicates that mismatching interactions may occur between plants and invading herbivores, and we further hypothesize that leaf tissue from Northern regions is less palatable for Southern communities of generalist herbivorous arthropods than foliage from the South (**Figure 1B**).

Thus, it is tempting to speculate that plants living at high latitudes might have a certain yet unknown intrinsic potential for defending themselves against pathogens and pests. Besides temperature and precipitation, the light conditions at high latitudes with extremely long daylight in summer may have a yet neglected role by causing a higher constitutive defense level.

## Jasmonates as key compounds in the regulation of induced plant defenses

**Figure 2:**
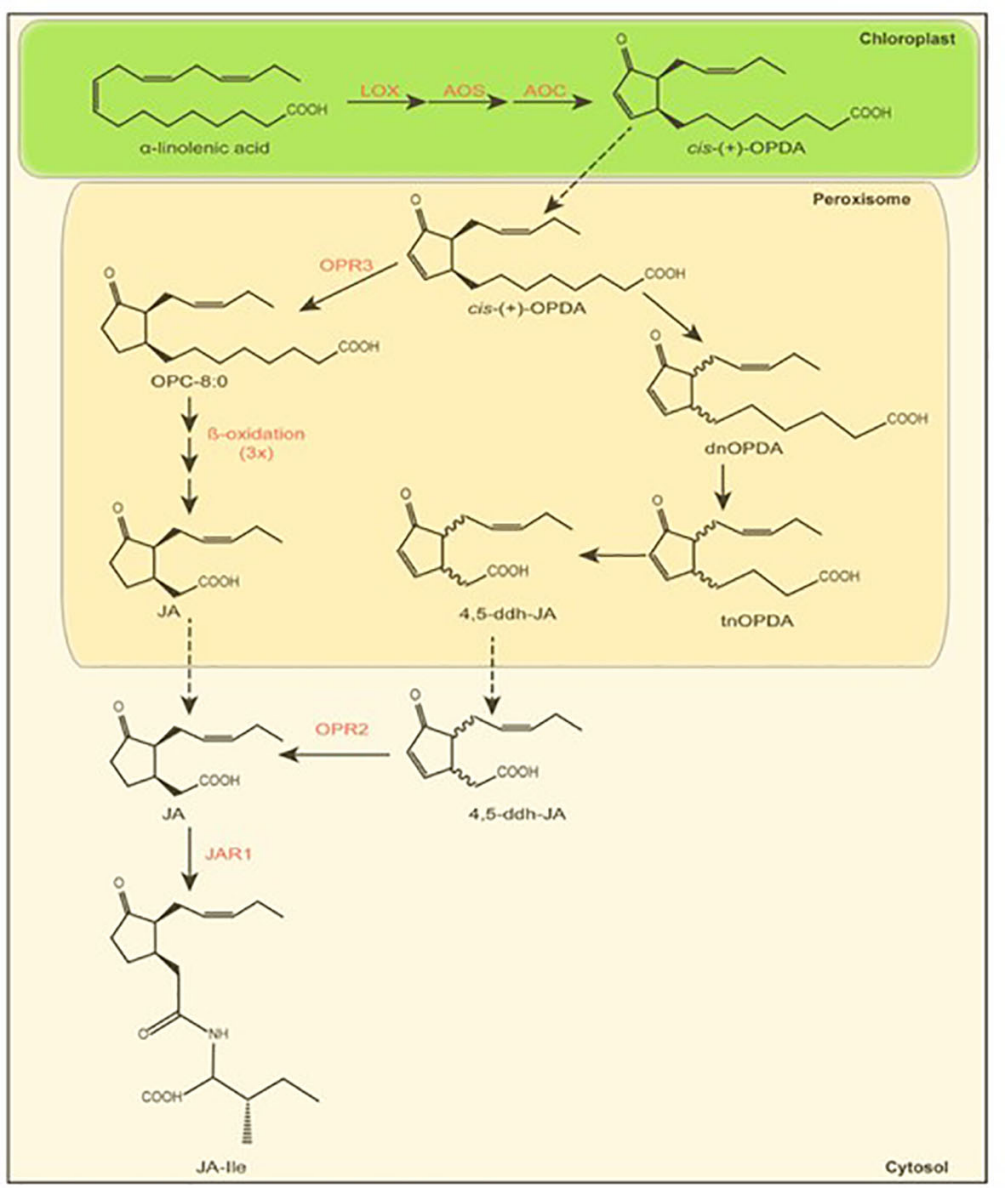
Jasmonate biosynthesis. Simplified scheme showing the generation of jasmonoyl-isoleucine (JA-Ile) via two alternative jasmonic acid (JA) pathways. Enzymatic reactions and corresponding enzymes are indicated in red. Transport between cell compartments is depicted by black dotted arrows. Abbreviations: LOX, Lipoxygenase; AOS, Allene Oxide Synthase; AOC, Allene Oxide Cyclase; OPDA, 12-oxo-phytodienoic acid; dnOPDA, dinor-OPDA; tnOPDA, tetranor-OPDA; 4,5-ddh-JA, 4,5-didehydro-JA; OPR, OPDA Reductase; OPC-8, 8-(3-oxo-2-(pent-2-enyl)cyclopentenyl)octanoic acid; JA, jasmonic acid; JAR, Jasmonate resistant; JA-Ile, jasmonoyl-isoleucine. Scheme modified after Chini et al. (2018) and Wasternack and Song (2017).

Jasmonates (JAs) are lipid-derived phytohormones that regulate a broad range of biological processes in plants, including plant growth, development, tolerance to abiotic stresses, and the production of secondary metabolites (Ueda et al., 2020). In particular jasmonic acid (**Figure 2**) is a well-known mediator of plant defense in response to wounding, herbivory (Howe and Jander, 2008; Koo et al., 2009), and pathogen infection (Glazebrook, 2005). The first steps of jasmonate biosynthesis (**Figure 2**) occur inside the chloroplast starting from α-linolenic acid and followed by a three-step enzymatic reaction, which is catalyzed by lipoxygenase (LOX), allene oxide synthase (AOS), and allene oxide cyclase (AOC). Thus, α-linolenic acid is converted into 12-oxophytodienoic acid (OPDA). Thereafter, OPDA is transported into the peroxisome and reduced by the OPDA reductase 3 (OPR3) into 8-(3-oxo-2-(pent-2-enyl)cyclopentenyl)octanoic acid (OPC-8:0) (Wasternack and Song, 2017) followed by three rounds of β-oxidation, ultimately synthesizing jasmonic acid. Alternatively, jasmonic acid can be formed through the intermediates dinor-12-oxophytodienoic acid, tetranor-12-oxophytodienoic acid, and 4,5-didehydrojasmonic acid after three β-oxidations, followed by OPR2-mediated reduction (Chini et al., 2018). Long time, jasmonic acid was thought to be the bioactive jasmonate. However, Staswick and Tiryaki (2004) and Fonseca et al. (2009) revealed that the isoleucine conjugate (JA-Ile) is the bioactive form. The production of JA-Ile in the cytoplasm is catalyzed by the jasmonic acid-amido synthetase JAR1 (JASMONATE RESISTANT 1; Staswick and Tiryaki, 2004).

In the absence of stress, only low levels of JA-Ile are present in the cell. In this situation, the Topless (TPL)-Novel Interactor of JAZ adapter proteins (NINJA)-JA-ZIM-domain (JAZ) complex represses MYC transcription factors, which are involved in the transcription of JAs-responsive genes. Upon stimuli such as herbivory or wounding, JA-Ile accumulates and binds to the receptor CORONATINE-INSENSITIVE1 (COI1) (Fonseca et al., 2009; Sheard et al., 2010). COI1 is part of the SCF complex, an E3 ubiquitin ligase (Devoto et al., 2002). Upon binding of JA-Ile, the SCFCOI1 complex ubiquitinates the jasmonate-ZIM-domain (JAZ) proteins, leading to their degradation by the 26S proteasome (Chini et al., 2007; Fernandez-Calvo et al., 2011). Consecutively, MYC transcription factors are released and lead to the expression of JAs-regulated defense- or stress-related genes.

## Jasmonates, light and phytochrome

Besides mechanical stress, JAs biosynthesis and signaling can be regulated by light through the phytochrome system (Kazan and Manners, 2011; Svyatyna and Riemann, 2012; Ballaré, 2014). A relationship between JAs metabolism/signal transduction and photoperception became obvious first in studies related to the photomorphogenesis of monocot seedlings in rice (*Oryza sativa*) (Riemann et al., 2003; Haga and Iino, 2004) and maize (*Zea mays*) (He et al., 2005). In rice, results indicated that JAs biosynthesis and signaling are necessary to obtain a complete photomorphogenic response in young, etiolated seedlings. Genetic evidence from mutants that were affected in JAs biosynthesis enzymes such as AOC (Riemann et al., 2013; Nguyen et al., 2020) or JAR1 (Riemann et al., 2008; Svyatyna et al., 2014) showed clear photomorphogenic phenotypes such as longer coleoptiles. However, there is also a feedback of JA on the phytochrome receptors and signaling hub. In JAs deficient rice coleoptiles, the light-dependent degradation of phytochrome A (phyA) is significantly decreased compared to the wild type (Riemann et al., 2009).

**Figure 3:**
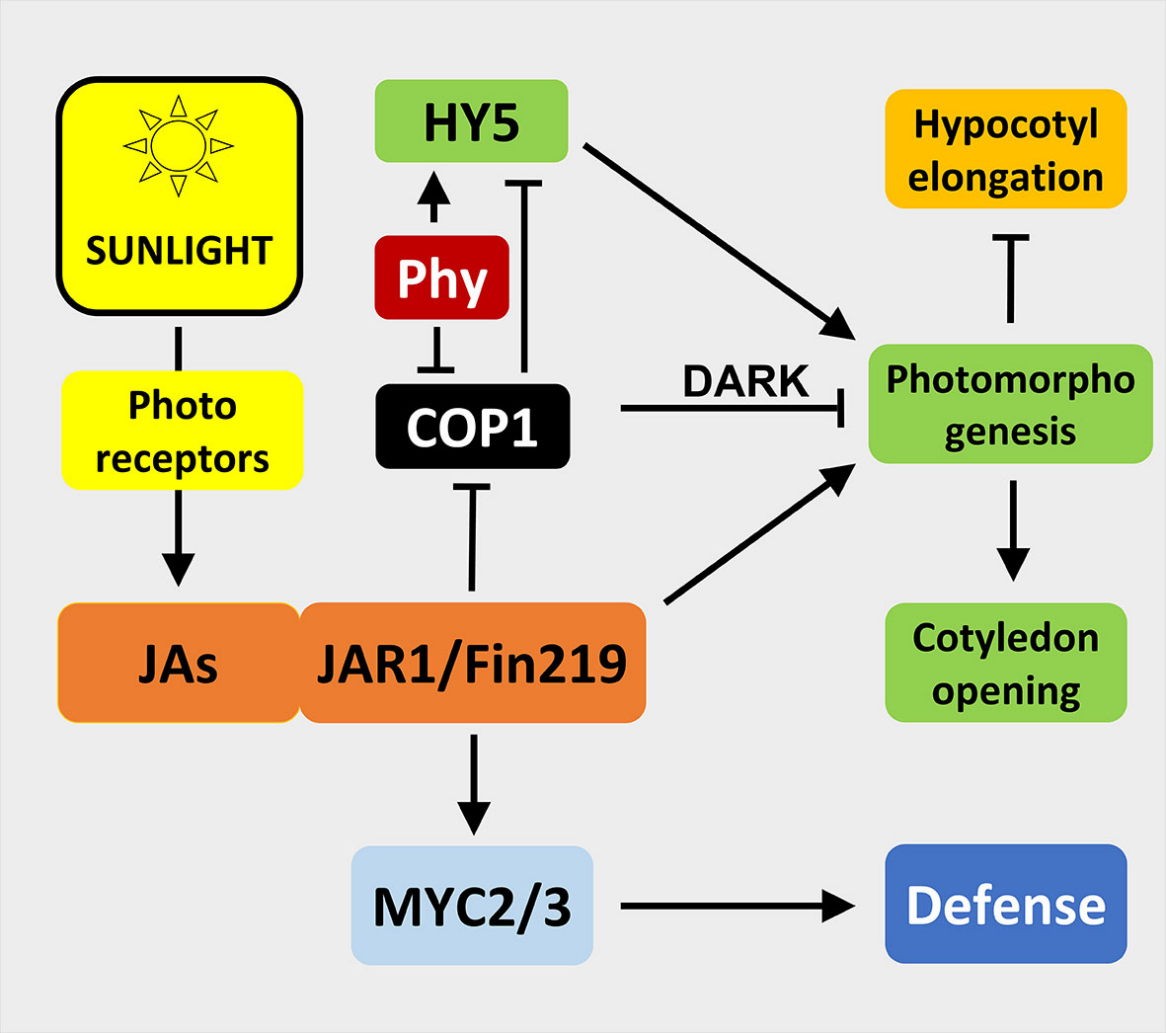
Illustration of interactions among sunlight, jasmonates, photomorphogenesis, and defense. Arrows imply activation, T-bars indicate inhibition of an interaction/process. JAs, jasmonates; JAR1/FIN219, Jasmonate resistant1/Far-red insensitive 219; COP1, Constitutive photo-morphogenic1, acts only in the dark on photomorphogenesis; Phy, Phytochrome; HY5, Elongated hypocotyl 5; MYC2/3, Transcription factors; photoreceptors include phytochrome, blue light, and UV-B receptors; sunlight represent R:FR >> 1. For details see text.

Similar mechanisms were found in other plants such as *Arabidopsis thaliana* (Chen et al., 2007; Robson et al., 2010). Here, recently a feedback mechanism of JAs on the phytochrome signaling machinery has been described: JAs inhibits CONSTITUTIVE PHOTO-MORPHOGENIC1 (COP1), a repressor of photomorphogenesis in the dark, and hence suppresses hypocotyl elongation and promotes cotyledon opening (Zheng et al., 2017). The Far-Red (FR) insensitive 219 (FIN219) line was demonstrated to be a suppressor of COP1 as well (Hsieh et al., 2000). Strikingly, *JAR1* has the same locus as *FIN219* (Staswick et al., 2002), which suggests that FIN219/JAR1 is an important regulator in modulating the integration of phytohormone-signaling through jasmonates and light signaling (Chen et al., 2007) (**Figure 3**).

Like in rice, JAs biosynthesis in Arabidopsis is induced by light (Yi et al., 2020). Photomorphogenesis of Arabidopsis is promoted via MYC2 and MYC3 transcription factors, representing positive regulators of the JAs response (Ortigosa et al., 2020). Both can activate the promoter of ELONGATED HYPOCOTYL 5 (HY5), a key transcription factor regulating photomorphogenesis. All these findings illustrate how closely JAs, light, and phytochrome signaling are linked in young seedlings (**Figure 3**). Thus, it is tempting to speculate that this holds true also for later stages in plant life. Indeed, it was found that a low R:FR ratio is characteristic for the shade avoidance response of plants, a typical phytochrome regulated process, which is induced in plants to escape from the shade and reach sunlight in order to find optimal conditions to perform photosynthesis. Further studies confirmed this discovery summarized in several review articles (Kazan and Manners, 2011; Ballaré, 2014; Fernández-Milmanda and Ballaré, 2021) and supported the trade-off between growth and defense hypothesis.

Very likely, additional photoreceptors (e.g. for blue and UV-B light) as well as additional plant hormones contribute to these responses (Fernández-Milmanda and Ballaré, 2021). In particular, gibberellic acid (GA) seems to be involved. The increased degradation of DELLA, a suppressor of GA signaling, led to more stable JAZ10 proteins thereby inhibiting JAs action (Yang et al., 2012; Leone et al., 2014). In summary, it is obvious that JAs assist light-dependent photomorphogenesis and attenuate dark-related growth promotion (**Figure 3**).

## Jasmonates, light and chemical defense

Many studies have shown that low R:FR ratios due to shade down-regulate defense responses in favor of growth (see: Ballaré, 2014). Plants grown in high density or under a low R:FR ratio display a partially impaired defense response against herbivorous insects, which was attributed to lower sensitivity to JAs (Moreno et al., 2009). It is evident that leaf tissue from plants grown in the shade is more favorable to herbivores, and shading increases infection by a range of pathogens (Roberts and Paul, 2006). Moreover, phyB inactivation leads to increased insect pest and pathogen susceptibility, which has been linked to reduced expression of defense-related chemical traits (Izaguirre et al., 2006; Moreno et al., 2009; Agrawal et al., 2012; Cargnel et al., 2014). However, if low R:FR or shade increase susceptibility of plants under attack, high R:FR or ambient light should do the opposite. This was demonstrated by Moreno et al. (2009) who showed that under FR light feeding herbivores (*Spodoptera frugiperda* larvae) performed better and gained more weight than under ambient light conditions. Moreover, herbivory is reduced in plants grown in full sunlight compared with plants grown in shade (Roberts and Paul, 2006; Schrijvers-Gonlag et al., 2020) (**Figure 3**). Many studies have been published concerning JAs-dependent control of metabolism (Mithöfer and Boland, 2012; Wasternack and Song, 2017; Savchenko et al., 2019), but whether such regulatory mechanisms are light-dependent and how light can influence the primary and secondary metabolism of plants through JAs and possibly other hormone pathways has rarely been investigated so far. A huge variety of regulatory mechanisms can be expected in the plant kingdom due to its rich and individually varied secondary metabolism. In snapdragon (*Antirrhinum majus*), the biosynthesis regulation of floral fragrance by light quality is mediated by JAs and calcium ions (Yang et al., 2022). In *Artemisia annua* a light and JAs-mediated control of artemisinin biosynthesis has been described (Fu et al., 2021). *Vitis vinfera* uses stilbenes as major defense compounds (Chong et al., 2009). It has been found that the application of methyl-jasmonate induced the production of stilbenes in grapevine suspension cells and that this accumulation of secondary metabolites could be further enhanced by red light (Tassoni et al., 2012). These few examples indicate the presence of regulatory mechanisms in different plant species and the potential of modulating the content of valuable defense-related compounds in plants depending on light quality and amount. However, it cannot be ruled out that herbivorous insects also adapt to the new environmental conditions and develop mechanisms that counteract JAs-mediated defense, for example through more efficient or novel effectors that suppress JAs synthesis

## Arctic summer light conditions cause elevated jasmonates and defense level

In summer, plants growing at high latitudes encounter very long photoperiods. Of course, in these latitudes the solar elevation angle is low, even in summer. This has an impact on the light quality. If the solar elevation angle is lower than 10°, also the R:FR ratio is lower than 1 (Mølmann et al., 2021). However, during the whole growth period from May to September, for the longest time of the days, the angle is higher than 10°. This supports our idea that Arctic summer day light conditions not only allow photosynthesis but also may cause higher resistance against attackers as supported by studies from Roberts and Paul (2006) and Schrijvers-Gonlag et al. (2020). The higher resistance might be due to intrinsic higher JAs levels. Based on the above, our hypothesis is as follows: Arctic summer light conditions cause elevated jasmonates and defense level.

**Figure 4:**
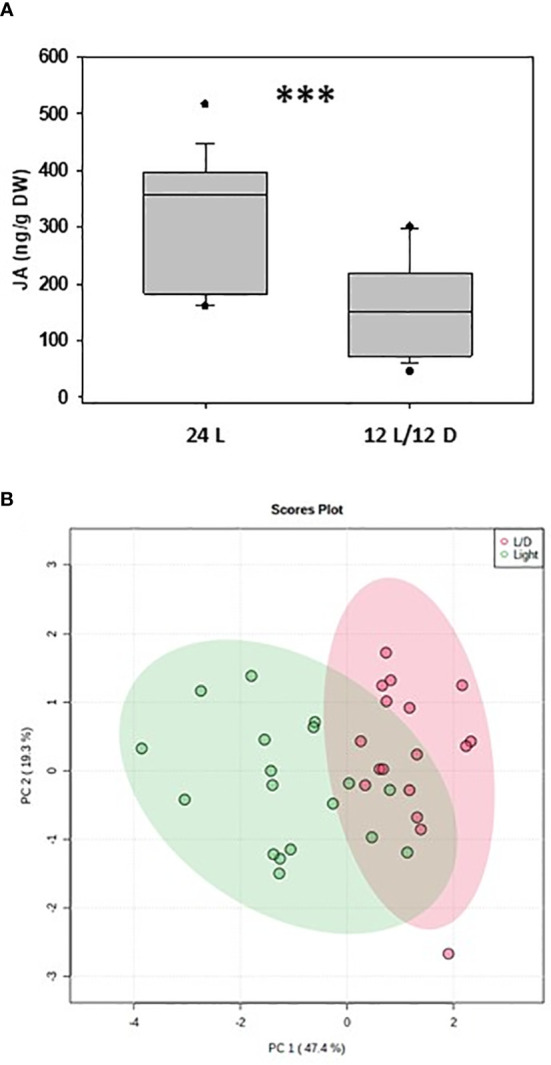
Phytohormones in bilberry (Vaccinium myrtillus) plants grown under different light regimes. **(A)** Accumulation of jasmonic acid (JA) in bilberry leaves grown for 14 days under 24 h (24 L) and 12 h (12 L/12 D) light regime and collected every 6 hrs, i.e. for the 12 L/12 D light regime two times each in the light and in the dark; ***: P < 0.001 (Welch-test). Phytohormone analysis was done according to Dávila-Lara et al. (2021). **(B)** Principal component analysis of amounts of various defense–related phytohormones (JA, JA-Ile, SA, IAA, ABA) accumulating under 12 h light (L/D) and 24 h light (Light) in bilberry leaves. PC, principal component (% of total variance); confidence area, 95% PCA analysis.

In order to test this hypothesis, we conducted a pilot study where naturally occurring bilberry (*Vaccinium myrtillus* L.) plants were collected in Northern Norway close to Tromsø (69° 29’ N) and grown under arctic (24 h light) and Central European (12 h light/12 h dark) light regimes. After two weeks, the leaf samples were collected every 6 hours in liquid nitrogen and stored at -80 °C. Eventually, samples were freeze dried, grinded and used for phytohormone analyses. We found that the levels of jasmonic acid (**Figure 4A**), abscisic acid, and auxin but not salicylic acid were significantly higher under 24 h light. A principal component analysis of the accumulation of these defense-related phytohormones revealed a partial separation between the two different light regimes (**Figure 4B**). These results are in accordance with earlier studies showing that increased light irradiation activates the synthesis of phenolic compounds in bilberry leaves, likely controlled by JA-mediated signaling (Jaakola et al., 2004; Benevenuto et al., 2019), and could at least partially explain the higher levels of phenolic compounds detected in bilberry leaves towards higher latitudes (Martz et al., 2010). A very recent experiment with *Chenopodium ficifolium* grown either under long-day (18 h light/6 h dark) and short-day (6 h light/18 h dark) regimes also showed that after three weeks the levels of JAs but also of abscisic and salicylic acids were significantly higher in long-day plants. The same was demonstrated for many stress-related genes (Gutierrez-Larruscain et al., 2022).

**Figure 5:**
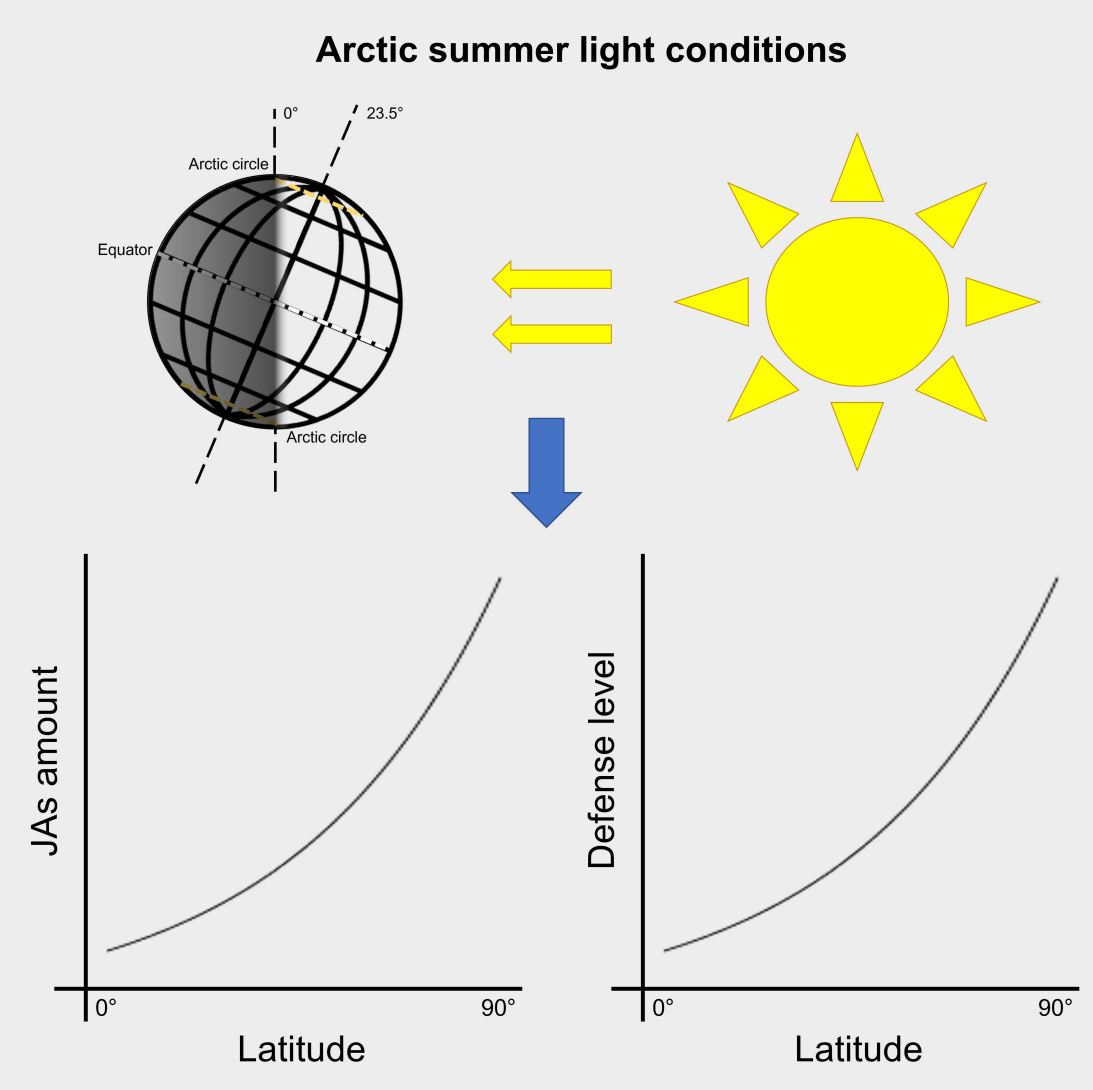
Model summarizing the suggested impact of arctic summer light conditions on jasmonates (JAs) and plant defense.

These findings suggest that at high latitudes light is an important factor for JAs accumulation and, consequently, affects JA-dependent downstream defenses (summarized in **Figure 5**). A systemically higher level of JAs very likely contributes to constitutive defenses directed against herbivory or any other stress-induced defense that is mediated *via* the jasmonate pathway, including the defense against nectrotrophic fungi. This might also be true for other phytohormones signaling pathways that directly or indirectly interact with JA signaling. Given that during evolution plants adapted to various light qualities and intensities, it is not surprising that light interacts with the action of phytohormones such as JAs that affect both development and defense. Our controlled pilot study was performed with local wild bilberry ecotype adapted to long day light conditions during the growth season. To further explore the response of this species to different day length conditions, controlled experiments with plants originating from different latitudinal locations will be performed. Additional, comprehensive studies to better understand the underlying molecular mechanisms in bilberry and other plants would lead to potential agricultural benefits for crops grown in the far North, which could become important in the future, especially under climate change conditions.

## Data availability statement

The raw data supporting the conclusions of this article will be made available by the authors, without undue reservation.

## Author contributions

Concept: AMi. CF, LJ, AMi performed the experiments. AMi and MR wrote the manuscript with contribution of all authors. All authors contributed to the article and approved the submitted version.

## Funding

AMr received a Martina Roeselova memorial fellowship. The Max Planck Society covered the open access fee.

## Acknowledgments

We thank the Max Planck Society. We also thank Michael Reichelt for phytohormone measurements. The staff of the Climate laboratory Holt at UiT The Arctic University of Norway is acknowledged for running the controlled growth experiment. AMr thanks Martina Roeselova memorial fellowship.

## Conflict of interest

The authors declare that the research was conducted in the absence of any commercial or financial relationships that could be construed as a potential conflict of interest.

## Publisher’s note

All claims expressed in this article are solely those of the authors and do not necessarily represent those of their affiliated organizations, or those of the publisher, the editors and the reviewers. Any product that may be evaluated in this article, or claim that may be made by its manufacturer, is not guaranteed or endorsed by the publisher.
